# The contractile vacuole in Ca^2+^-regulation in *Dictyostelium*: its essential function for cAMP-induced Ca^2+^-influx

**DOI:** 10.1186/1471-213X-6-31

**Published:** 2006-06-20

**Authors:** Dieter Malchow, Daniel F Lusche, Christina Schlatterer, Arturo De Lozanne, Annette Müller-Taubenberger

**Affiliations:** 1 Department of Biology, University of Konstanz, D-78457 Konstanz, Germany; 2 Section of Molecular Cell Developmental Biology, University of Texas at Austin, Austin, Tex 78712, USA; 3 MaxPlanckInstitute for Biochemistry, D-82152 Martinsried, Germany; 4 WM Keck Research Facility, Department of Biological Sciences 014 BBE Iowa City, IA 52242, USA; 5 Institute for Cell Biology (ABI), Ludwig Maximilians University München, D-80336 München, Germany

## Abstract

**Background:**

cAMP-induced Ca^2+^-influx in *Dictyostelium *is controlled by at least two non-mitochondrial Ca^2+^-stores: acidic stores and the endoplasmic reticulum (ER). The acidic stores may comprise the contractile vacuole network (CV), the endosomal compartment and acidocalcisomes. Here the role of CV in respect to function as a potential Ca^2+^-store was investigated.

**Results:**

Dajumin-GFP labeled contractile vacuoles were purified 7-fold by anti-GFP-antibodies in a magnetic field. The purified CV were shown for the first time to accumulate and release Ca^2+^. Release of Ca^2+ ^was elicited by arachidonic acid or the calmodulin antagonist W7, the latter due to inhibition of the pump. The characteristics of Ca^2+^-transport and Ca^2+^-release of CV were compared to similarly purified vesicles of the ER labeled by calnexin-GFP. Since the CV proved to be a highly efficient Ca^2+^-compartment we wanted to know whether or not it takes part in cAMP-induced Ca^2+^-influx. We made use of the LvsA^-^-mutant expected to display reduced Ca^2+^-transport due to loss of calmodulin. We found a severe reduction of cAMP-induced Ca^2+^-influx into whole cells.

**Conclusion:**

The contractile vacuoles in *Dictyostelium *represent a highly efficient acidic Ca^2+^-store that is required for cAMP-induced Ca^2+^-influx.

## Background

The contractile vacuole (CV) network of *Dictyostelium *consists of tubes and bladders. It transiently fuses with the plasma membrane to expel water and ions and thereby serves as an efficient osmoregulatory organelle [1,2]. The CV-system is also assumed to be involved in Ca^2+^-transport since it contains a PMCA-type Ca^2+^-ATPase (PAT1), calmodulin [3] and a vacuolar proton pump that establishes a proton gradient for Ca^2+^-transport [4]. PAT1 is localized to the CV and the plasma membrane and is upregulated under conditions of Ca^2+^-stress [5]. Downregulation of PAT1 by antisense RNA reduced vesicular Ca^2+^-uptake.

We are interested in the characterization of Ca^2+^-stores that are involved in cAMP-induced Ca^2+^-influx. Previously, it has been shown that acidic Ca^2+^-stores and an IP_3_-sensitive store participate in this regulation [6-11]. Acidic means that the stores are equipped with a V-type H^+^-ATPase. Acidic vesicular Ca^2+^-stores in *Dictyostelium *comprise the CV-system, endosomes and acidocalcisomes [12,13]. In the present study we focus on the contribution of the CV-system to intracellular Ca^2+ ^regulation.

It has been shown previously that GFP-tagged dajumin labels the entire CV whereas the endosomal compartments are devoid of the label [14]. By contrast, drainin, a peripheral membrane protein involved in discharge of the bladder, is found only on the bladder [15]. We used dajumin-GFP expressing cells to obtain a fraction enriched in CV membranes and used this fraction to measure Ca^2+^-transport. Ca^2+^-transport activity increased with enhanced purity of the CV. We also employed a LvsA minus strain which lacks the gene for the protein large volume sphereA (*lvsA*). Besides its involvement in cytokinesis [16] it is known that the LvsA-protein is localized to the CV. This association with the CV occurs only during the discharge phase of the vacuole. In the lvsA mutant calmodulin was lost from the CV-membranes and the CV became disorganized, unable to discharge its contents [17]. We found that vesicular Ca^2+^-transport in the lvsA-mutant was diminished and that cAMP-induced Ca^2+^-influx was drastically reduced, indicating that functional CV are absolutely required for the cAMP-dependent Ca^2+^-influx.

## Results

### Distribution of CV in vesicular fractions

**Figure 1:**
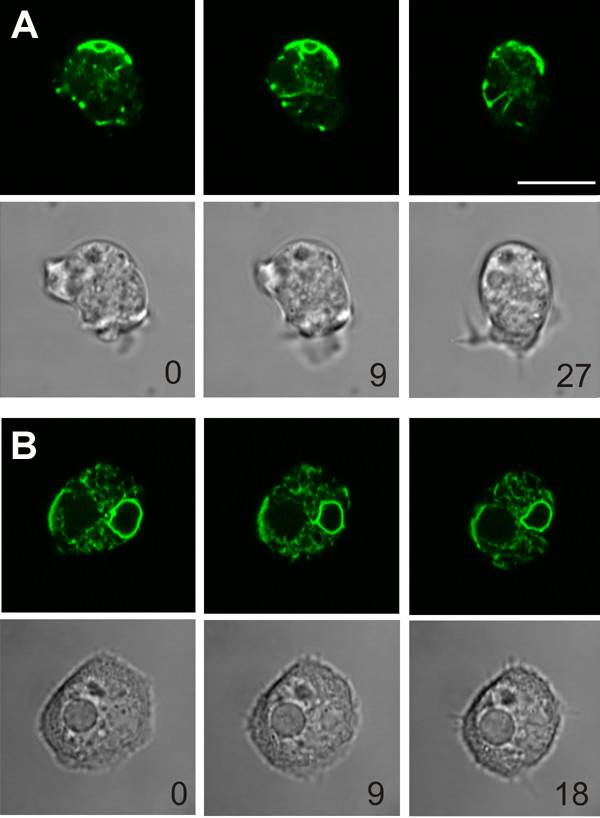
***Dictyostelium *wild-type Ax2 cells expressing dajumin-GFP or calnexin-GFP**. (A): The contractile vacuole system of a *Dictyostelium *cell is visualized by expression of dajumin-GFP using live cell confocal microscopy (upper panels). Dynamics of the bladder is indicated by formation of irregular ventricles and ducts. Lower panels show the corresponding bright field images. (B): A *Dictyostelium *cell expressing calnexin-GFP to visualize the endoplasmic reticulum is shown in the upper panels. Note that the perinuclear region is intensively labeled as observed previously for calnexin-GFP and antibody-stained preparations, whereas a vacuole in the left part of the cell is devoid of any label. Time of depicted frames is indicated in seconds. Bar corresponds to 10 μm.

We used differentiated cells 4 to 5 hrs after starvation for preparation of Ca^2+^-transporting vesicles because cAMP-induced Ca^2+^-influx is present at that time and endosomal content is low (see below). Cells labeled with dajumin-GFP as a marker for the CV-system or with calnexin-GFP cells as a marker for the endoplasmic reticulum (ER) are shown in Figure 1. Dajumin-GFP allows to visualize the dynamics of the bladder by formation of irregular ventricles and ducts (A). The ER is prominently labeled in the perinuclear region and close to the plasma membrane (B). The cells were lysed by passage through nuclepore filters. Vesicular fractions were separated by differential centrifugation and assayed for Ca^2+^-transporting activity. The dajumin-GFP label was detected in vesicular fractions with the majority being present in the fast sedimenting fraction P_0 _(Table 1). By contrast, most of the ER occurred in P_1_, whereas the lightest fraction P_2 _contained only a quarter of both organelles (Table 1). Plasma membranes, as shown previously, sedimented in P_1 _[18]. Ca^2+^-transport activity was strikingly concentrated in P_0_. The presence of endosomes was measured with RITC-dextran. In two independent experiments 38 ± 6% of the label was associated with P_0_, 62 ± 6% with P_1 _and none was present in P_2_. However, the amount of endosomes of 4 hour starved cells was low and barely detectable. This result is expected since the cells develop in the absence of nutrients. If endosomes accumulate Ca^2+ ^their contribution to Ca^2+^-transport was therefore considered to be insignificant under the present experimental conditions.

**Table 1:** Characterization of crude vesicular fractions. Vesicles were obtained by differential centrifugation of lysed cells as described in Methods. The amount (percent) of CV (± S.D.) in each fraction was determined according to the dajumin-GFP label present, while that of the ER by the presence of calnexin-GFP label. Total Ca^2+^-transport activity obtained from 4 × 10^8 ^cells is shown for each fraction. Data are means ± SD. The number of independent experiments is given in brackets.

Fraction	Sedimentation	CV	ER	Ca^2+^-uptake	
				
	(g)	%	%	nmol	%
P_0_	3.800	42.4 ± 4.0 (4)	33.5 ± 3.7 (3)	205 ± 42 (3)	67
P_1_	12.000	33.6 ± 7.2 (4)	43.0 ± 2.8 (3)	76 ± 24 (3)	25
P_2_	40.000	24.0 ± 10.6 (4)	23.5 ± 1.3 (3)	25 ± 6 (3)	8

### Purification of CV with magnetic antibody-beads

Since P_0 _was the richest source of CV we incubated P_0 _isolated from dajumin-GFP labeled cells with anti-GFP-magnetic beads and loaded the mixture onto a column. During loading and washing the beads were retained on the column by applying a magnetic field. Elution was performed in its absence. As shown in Table 2 almost all of the dajumin-label was present in the eluate (P_0_E) as well as most of Ca^2+^-uptake. The flow through (P_0_F) still contained some Ca^2+^-transport activity the percentage of which, however, exceeded only slightly the remaining dajumin-GFP-label.

**Table 2:** Purification of CV and ER fractions by antibody. Dajumin-GFP labeled CV present in P_0 _were bound to anti-GFP-microbeads and separated from the unbound vesicles in a magnetic field as detailed in Methods. P_0_F represents the flow through, P_0_E is the eluate containing CV. Calnexin-GFP labeled ER present in P_1_E was purified in the same way. P_1_F: flow through, P_1_E: eluate containing ER. As described in Methods, the measured values of Ca^2+^-uptake were corrected for the same initial [Ca^2+^]_ev_. Data are means ± SD. The number of independent experiments is given in brackets.

Fraction	GFP-label %	Ca^2+^-uptake %	Specific activity of Ca^2+^-ATPase (%)
P_0_F (Dajumin)	12.4 ± 3.9 (3)	15.6 ± 9.7 (3)	18 ± 5.4 (3)
P_0_E (Dajumin)	87.6 ± 3.9 (3)	84.4 ± 9.7 (3)	109 ± 9.3 (3)

P_1_F (Calnexin)	13.8 ± 4.2 (4)	13.4 ± 8.3 (4)	8.1 ± 6.3 (4)
P_1_E (Calnexin)	86.2 ± 4.2 (4)	86.6 ± 8.3 (4)	87 ± 66 (4)

The selectivity of the GFP-antibody beads for GFP was tested by assaying P_0 _from unlabeled Ax2-cells, the parent strain of dajumin-GFP expressing cells, for binding to the anti-GFP-beads. In three independent experiments 54 ± 31 μg (n = 6) protein was associated with P_0_E in the controls whereas 192 ± 60 μg (n = 6) protein was present in P_0_E from dajumin-GFP labeled cells. This result shows that the GFP-antibody specifically targets the GFP-tagged protein and that 28% of unspecific binding does occur.

In order to avoid contamination of P_0 _and subsequent fractions with plasma membranes due to possible association of plasma membranes and CV by cortical actin filaments we preincubated the cells (all experiments of Table 2) with latrunculin B. Latrunculin B, a unique marine toxin, inhibits actin polymerization and disrupts microfilament organization [19]. In the presence of latrunculin B the cells rounded up and were completely immotile before they were pressed through the filters. The characteristics of Ca^2+^-transport, its inhibition by drugs as well as Ca^2+^-release in P_0_E were in the same range as in preparations performed without latrunculin B treatment. Latrunculin B treatment reduced the protein content of P_0_E by a factor of 1.26 but there was no statistically significant difference in the specific activity of the Ca^2+^-ATPase.

**Figure 2:**
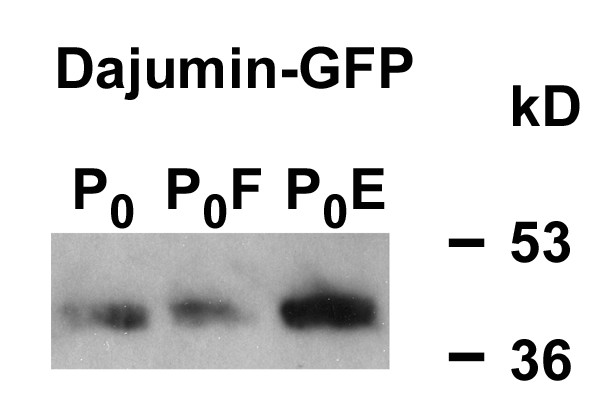
**Enrichment of V-type H^+^-ATPase in P_0_E**. Western blot analysis with antibodies to V-type H^+^-ATPase was carried out as described in Methods using fractions of cells expressing dajumin-GFP. 7 μg of protein per lane of each fraction were applied.

A specific marker for the CV as compared to the plasma membrane is the V-type H^+^-ATPase. Antibodies to the 37/42 kDa subunit revealed that the proton pump was markedly enriched in P_0_E (Fig. 2). A mitochondrial contribution to Ca^2+^-fluxes was prevented by inclusion of mitochondrial inhibitors in all vesicular Ca^2+^-transport measurements.

### Purification of ER with magnetic antibody-beads

Since P_1 _was the richest source of ER (Table 1) we incubated P_1 _isolated from calnexin-GFP labeled cells with anti-GFP-magnetic beads and separated ER-vesicles from other vesicles by a magnetic field. As shown in Table 2 almost all of the calnexin-GFP-label was found in the eluate (P_1_E) as well as most of Ca^2+^-uptake activity. The flow through (P_1_F) also contained some Ca^2+^-transport activity.

The results of Table 2 thus show that by using the anti-GFP-antibody beads a 6-7-fold enrichment of either CV or ER can be obtained. Furthermore, both fractions enriched either in CV or ER vesicles, displayed a similar enhancement of Ca^2+^-transport. This result demonstrates that vesicles of the CV of *Dictyostelium *are functional in Ca^2+^-homeostasis.

### Properties of CV and ER fractions with respect to Ca^2+^-regulation

**Figure 3:**
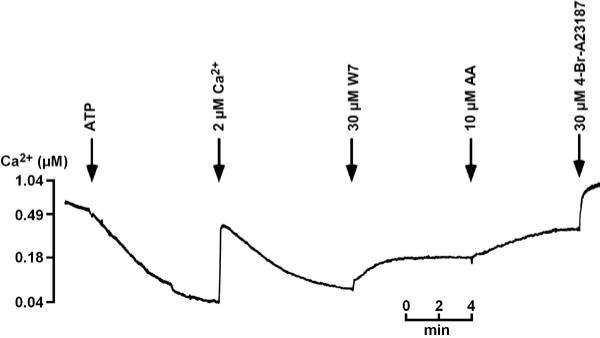
**Ca^2+^-uptake and Ca^2+^-release in P_0_E**. P_0_E was obtainedby GFP-antibody purification. Ca^2+^-uptake was induced by 0.4 mM ATP and measured with Fluo-3 as described in Methods. A Ca^2+^-calibration pulse was followed by further Ca^2+^-uptake. W-7 and AA induced Ca^2+^-release, 4-BrA23187 elicited further Ca^2+^-release. One out of three independent experiments is shown.

Since both purifications had given a strong enrichment of the GFP-label as well as in Ca^2+^-transport in the eluate (84–87%) and since in control experiments 28% unlabeled protein was associated with the eluate, we decided to test for the properties of CV and ER with respect to Ca^2+^-transport and Ca^2+^-release. As shown in Figure 3 P_0_E, the eluate of dajumin-GFP-labeled cells containing CV, transported Ca^2+ ^efficiently. Note, that the slope is fairly steep and uptake of Ca^2+ ^is settled in about 8 min. In three independent experiments we found that starting at an extravesicular Ca^2+^-concentration ([Ca]^2+^_ev_) of 0.53 ± 0.02 μM uptake ceased to 0.10 ± 0.05 μM [Ca]^2+^_ev _in the presence of 32 ± 12 μg protein. In agreement with previous results obtained for acidic Ca^2+^-stores the calmodulin antagonist W7 as well as arachidonic acid (AA) caused Ca^2+^-release in P_0_E. The response to W7 (30–100 μM) amounted to 21.4 ± 10.2 nmol Ca^2+^/mg protein (n = 4) and to AA (10–20 μM) 23.9 ± 7.2 nmol Ca^2+^/mg protein (n = 4).

**Figure 4:**
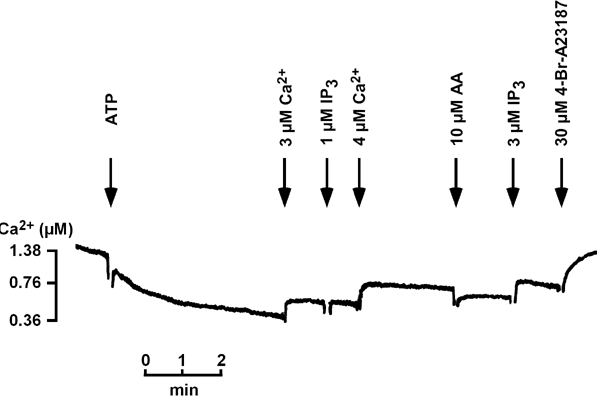
**Ca^2+^-uptake and Ca^2+^-release in P_1_E**. P_1_E was obtainedby GFP-antibody-purification. Ca^2+^-measurements were performed as described in the legend to Fig. 1. One out of three independent experiments is shown.

In Figure 4 Ca^2+^-uptake by P_1_E, the eluate of calnexin-GFP-labeled cells containing ER, is shown. In contrast to P_0_E P_1_E was less responsive to AA. In three out of four experiments no Ca^2+^-release was measured. The one that responded to AA had a lower purification factor with respect to GFP fluorescence compared with the other three, 3.8 versus 7.8 ± 0.96 (n = 3). IP_3_, most of the times, was inefficient to elicit Ca^2+^-release. In general, Ca^2+^-uptake was slower in P_1_E as compared to P_0_E starting at 0.56 ± 0.15 μM [Ca]^2+^_ev _and ceased to extravesicular Ca^2+^-concentrations of about 0.36 ± 0.08 μM in the presence of 27 ± 12 μg protein (n = 4), indicating that the CV is more relevant in maintaining a basal cytosolic Ca^2+^-concentration of 50 to 60 nM [10] than the ER.

In addition, we tested for inhibition of Ca^2+^-uptake. Table 3 shows that concanamycin A (CMA), a powerful inhibitor of V-type H^+^-ATPases [20,21] inhibited P_0_E as expected for a compartment that uses a proton gradient for Ca^2+^-transport and a Ca^2+^-transport ATPase PAT1. Under our experimental conditions BHQ, an inhibitor of Ca^2+^-transport ATPases, inhibited Ca^2+^-uptake only weakly despite its reported property to inhibit cAMP-induced Ca^2+^-influx in *Dictyostelium *[22,23]. BHQ was more efficient in inhibiting Ca^2+^-uptake into the ER-fraction. The latter was also sensitive to thapsigargin (Tg) a blocker of SERCA-type Ca^2+^-ATPases. In both fractions, P_0_E and P_1_E, Ca^2+^-uptake was inhibited by low concentrations (30–60 μM) of Sr^2+ ^by about 50%.

**Table 3:** Inhibition of Ca^2+^-uptake activity. The purified CV-fraction P_0_E and the purified ER-fraction P_1_E were tested for sensitivity of Ca^2+^-uptake to Tg (22 μM), BHQ (200 μM), CMA (40 μM) and Sr^2+ ^(30–60 μM) as indicated. nd: not determined. Data are means ± SD. The number of independent experiments is given in brackets.

Fraction	Tg %	BHQ %	CMA %	Sr^2+ ^%
P_0_E (CV)	nd	25 ± 13 (3)	57 ± 6 (4)	52 ± 13 (3)
P_1_E (ER)	45 ± 23 (4)	72 ± 4 (3)	nd	43 ± 6 (4)

### Is the CV involved in cAMP-induced Ca^2+^-influx?

Given our results that the CV is a Ca^2+^-store that transports Ca^2+ ^most efficiently at low Ca^2+^-concentrations we wanted to know whether the store is also involved in receptor-mediated Ca^2+^-flux. Although it was previously shown that NBD-Cl and CMA inhibited the cAMP mediated Ca^2+^-influx [7,22] these results only proved that acidic stores as a whole were involved. We made use of the LvsA^-^-mutant whose CV-function is abolished with respect to fusion of CV with the plasma membrane and is disturbed by a loss of calmodulin. Previously, we have shown that the calmodulin antagonist W7 inhibits Ca^2+^-transport of acidic Ca^2+^-stores [24]. A localized loss of calmodulin confined to the CV would have the same consequences. Therefore, it was interesting to know whether or not Ca^2+^-influx was also disturbed in the mutant. As shown in Table 4 there was no receptor-mediated Ca^2+^-influx in the LvsA minus strain at physiological cAMP-concentrations (0.1–10 μM) in contrast to the parent strain DH1. Only higher concentrations of cAMP yielded Ca^2+^-influx that was reduced as compared to DH1 (Table 4).

**Table 4:** Stimulus-induced Ca^2+^-influx in LvsA^-^and DH1. Ca^2+^-influx was determined with a Ca^2+^-sensitive electrode before and after addition of cAMP or AA at an extracellular Ca^2+^-concentration of 2–3 μM. Data are means ± SD. The number of independent experiments is given in brackets.

	Ca^2+^-influx pmol/10^7 ^cells	
Stimulus (μM)	LvsA^-^	DH1
cAMP 0.1	< 0.1 (7)	nd
1	< 0.1 (7)	100 ± 59 (3)
10	1 ± 5 (9)	203 ± 107 (3)
20 – 50	50 ± 1 (2)	nd
AA 10	< 0.1 (7)	70 ± 13 (3)

Besides cAMP, arachidonic acid (AA) was shown to elicit Ca^2+^-influx. AA acts by eliciting Ca^2+^-release from Ca^2+^-stores which is thought to induce a capacitative Ca^2+^-influx [7,23]. In the LvsA minus mutant AA did not evoke Ca^2+^-influx in contrast to the parent strain (Table 4). Instead Ca^2+^-efflux occurred. In seven independent experiments 6–10 μM AA induced an efflux of Ca^2+ ^of 276- ± 153 pmol/10^7^cells.

Analysis of vesicular Ca^2+^-transport in the mutant revealed that transport activity was present in all three fractions of P_0_, P_1 _and P_2 _albeit at a somewhat lower extent than in Ax2 (Table 5). Therefore, the small amount of cAMP-induced Ca^2+^-influx observed in the LvsA minus strain cannot be due to the lack of vesicular Ca^2+^-transport in the mutant.

**Table 5:** Vesicular Ca^2+^-transport of LvsA^-^. The specific Ca^2+^-transport activity of vesicular fractions of LvsA^- ^and Ax2 is shown. Data are means ± SD. The number of independent experiments is given in brackets.

	Ca^2+^-uptake nmol/mg	
	LvsA^-^	Ax2
P_0_	42 ± 19 (5)	68 ± 9.8 (3)
P_1_	29 ± 17 (3)	36 ± 7.9 (3)
P_2_	15 ± 3 (3)	23 ± 2 (3)

## Discussion

In this study we have shown that one of the acidic compartments of *Dictyostelium*, the CV-system, is required for cAMP-induced Ca^2+^-influx in intact cells and that isolated CV-vesicles mediate an essential part of Ca^2+^-transport as well as Ca^2+^-release in response to AA. Previously, it was only known that acidic Ca^2+^-stores were involved. The use of dajumin-GFP-labeled CV allowed a 7-fold enrichment of CV by a specific method utilizing antibodies directed against the GFP-tag. Ca^2+^-transport was enhanced 5- to 6-fold. This result is due to Ca^2+^-transport activity of another Ca^2+^-store that was retained in the flow through (P_0_F). Enriched CV displayed a potent and efficient Ca^2+^-transport that resulted in a low basal extravesicular Ca^2+ ^concentration. Moreover, the Ca^2+ ^taken up could be released by the calmodulin antagonist W-7 and by AA. Both compounds have been shown to be potent regulators of the acidic Ca^2+^-stores [23,24]. In addition, Ca^2+^-uptake was sensitive to CMA, an inhibitor of V-type H^+^-ATPases. Moreover, the V-type proton pump was concentrated in P_0_E. A similar enrichment was also obtained for the ER using calnexin-GFP-labeled cells. These Ca^2+^-stores differed from those of the CV by a greater sensitivity to BHQ, a lower responsiveness to AA and a higher basal extravesicular Ca^2+^-concentration following Ca^2+^-uptake.

In order to examine whether the Ca^2+^-transport occurring in CV is also involved in cAMP-mediated Ca^2+^-influx we made use of a mutant, LvsA minus. In this mutant the CV becomes disorganized during the discharge phase of the vacuole and calmodulin dissociates from the CV-membranes. The CV can swell but seems unable to discharge. The LvsA-protein that belongs to the family of BEACH-proteins transiently binds to the CV. We found that the mutant was strongly impaired in receptor-mediated Ca^2+^-influx when physiological cAMP-concentrations of 0.1–1 μM and even higher concentrations of 10 μM cAMP were applied. A significant Ca^2+^-influx was apparent only at elevated cAMP concentrations of 20 to 50 μM. Even so, influx was inhibited by about 75% as compared to the parent strain. Other compounds that induce Ca^2+^-influx in *Dictyostelium *are long chain fatty acids like AA. They act independently of the cAMP-receptor and of cell differentiation [7]. Similarly to cAMP, AA did not cause Ca^2+^-influx in the LvsA-minus-strain, instead Ca^2+^-efflux occurred. These results demonstrate that in order to activate capacitative Ca^2+^-influx an intact CV-system is absolutely necessary.

### What is the role of the LvsA-protein in Ca^2+^-influx?

The function of the LvsA-protein in *Dictyostelium *is not yet known. BEACH-proteins belong to several classes and only a few proteins have been characterized. The FAN-protein mediates TNF-activation via the MAP-kinase pathway [25]. Neurobeachin conveys anchoring of the regulatory subunit of protein kinase A (PKA) [26]. In both cases, the BEACH-protein participates in signal transduction that could lead to altered Ca^2+^-regulation in *Dictyostelium*. Elevated cAMP-levels in *Dictyostelium *were shown to affect both, basal [Ca^2+^]_i _and stimulus-induced [Ca^2+^]_i_-transients [27].

Apart from this, it was shown that the loss of the LvsA-protein was accompanied by a loss of calmodulin from the CV-membranes. Since we have shown that the calmodulin antagonist W7 inhibits Ca^2+^-transport of acidic Ca^2+^-stores [24] we conclude that Ca^2+^-uptake by the CV is inhibited in the absence of calmodulin. This could be an explanation of the severe reduction of cAMP-mediated Ca^2+^-influx measured in the LvsA^-^-mutant strain.

## Conclusion

Among the acidic Ca^2+^-stores the contractile vacuole represents an efficient Ca^2+ ^transport organelle that sequesters Ca^2+ ^to low extravesicular Ca^2+ ^concentrations and releases Ca^2+ ^in response to arachidonic acid. Furthermore, it is essential for the occurrence of receptor-mediated Ca^2+ ^influx to physiological concentrations of cAMP.

## Methods

### Chemicals

N-(6-aminohexyl)5-chloro-1-naphthalene sulfonamide (W-7) 2,5-di(tert-butyl)-1,4-hydroquinone (BHQ) and concanamycin A (CMA) were from Fluka (Buchs, Switzerland). Fluo-3 was obtained from MobiTec (Göttingen, Germany), inositol 1,4,5-triphosphate and thapsigargin from Alexis (Grünberg, Germany) and arachidonic acid from Sigma (München, Germany). The μMACS GFP-tagged protein isolation kit was purchased from Miltenyi Biotec (Bergisch Gladbach, Germany).

### Cells and culture

The strains were grown in liquid culture as discribed by Sonnemann et al. [28]. The dajumin-GFP [14] and calnexin-GFP [29] strains were raised in the presence of 50 μg/ml G418. The LvsA-minus-strain [16] was grown with 100 μg/ml ampicillin and 60 μg/ml streptomycin and its parent strain DH1 additionally with 20 μg/ml uracil. Differentiation was induced by washing the cells twice in ice cold Sørensen phosphate buffer (17 mM KH_2_/Na_2_H, PO_4_, pH 6.0). Cells were shaken on a rotary shaker at 23°C, 150 rpm at 2 × 10^7 ^cells/ml until use.

#### Preparation of vesicles

20 ml 2 × 10^7^cells/ml were shaken in Sørensen phosphate buffer containing 5 mM EGTA for 2–4 h, washed once in ice-cold 20 mM Hepes buffer, pH 7.2, resuspended at 2 × 10^8 ^cells/ml, and lysed by passage through nuclepore filters. Immediately, 3% sucrose, 50 mM KCl, 1 mM MgCl_2_, 10 μg/ml leupeptin, 1 μg/ml aprotinin and 1.25 mM dithiothreitol were added (final concentration). After centrifugation for 5 min at 150 × g in order to remove unbroken cells, the supernatant was further fractionated by centrifugation for 5 min at 3800 × g. The sediment (P_0_) was resuspended in 1 ml 10 mM Tris buffer, pH 7.8, 3% sucrose, 50 mM KCl, 1 mM MgCl_2_, 20 μg/ml leupeptin and 2 μg/ml aprotinin (TKS-buffer). The supernatant was centrifuged at 12.000 × g for 20 min. The sediment (P_1_) was resuspended in 0.9 ml TKS-buffer. The supernatant was further centrifugated at 40.000 × g for 30 min. The fluffy sediment was resuspended in about 0.6 ml TKS-buffer and designated P_2_.

### Purification of vesicles with anti-GFP-magnetic microbeads

400 μl P_0 _suspended in 10 mM Tris-buffer, pH 7.8, 50 mM KCl, 20 μg/ml leupeptin and 2 μg/ml aprotinin (TK-buffer) was incubated for 30 min at 4°C with 100 μl anti-GFP-microbeads and then transferred to a Mini MACS-separation column exposed to a magnetic field. The column had been rinsed with 500 μl TK-buffer. The flow through was centrifuged for 5 min at 3.800 × g. The sediment was resuspended in 150 μl of TKS-buffer and designated P_0_F. In the meantime the column was washed with 500 μl TK-buffer, 500 μl TK-buffer containing 0.1 M NaCl and 500 μl TK-buffer containing 0.3 M NaCl. In case the column was running inefficiently the contents were transferred to a new column by pressing 500 μl TK-buffer through the old column in the absence of a magnetic field onto the new column positioned in the field. The final elution was performed in the same way after the column had been washed free of salt with TK-buffer. The washing solutions usually contained negligible amounts of the GFP-label. The purified eluate was centrifuged for 5 min at 3.800 × g, resuspended in about 350 μl TKS-buffer and designated P_0_E.

In most experiments the cells were treated with 8 μM latrunculin B for 20 min before lysis to disrupt F-actin filaments. Under this condition the cells rounded up and became immotile. Nonetheless, the dajumin-GFP-label remained associated with P_0 _and purification did not change significantly.

### Ca^2+^-transport

Ca^2+^-transport was measured essentially as described [23]. In brief, about 60 μl fraction was added to 10 mM Hepes, pH 7.2, 50 mM KCl, 3% sucrose, 6 μg/ml antimycin A, 6 μg/ml oligomycin A, 100 μM NaN_3_, 2 mM MgCl_2_, and about 0.5 μM Fluo-3 in a total volume of 1 ml. Ca^2+^-uptake was initiated by addition of 400 μM ATP. Fluo-3 fluorescence was monitored at 505 nm excitation and 526 nm emission with a fluorimeter (Perkin Elmer 650-10S, Überlingen, Germany). Since [Ca^2+^]_ev _at the beginning of the experiment is not known and Ca^2+^-ATPase activity is increasing with increasing Ca^2+^-concentrations the results shown in Table 2 were corrected for the same initial [Ca^2+^]_ev _in order to relate the outcome of different experiments to each other. This was done by using a calibration curve at different [Ca^2+^]_ev _obtained with either P_0 _or P_1_, respectively, and adjusting the measured value to a [Ca^2+^]_ev _of 1 μM. The Ca^2+^-ATPase activities of both, P_0 _and P_1_, increased by a factor of about 3.3 from 0.5 μM to 1.5 μM [Ca^2+^]_ev_. Statistical data are expressed throughout as the mean ± S.D.

### Immunoblotting

Seven microgram of protein per lane were resolved by SDS-PAGE in 12.5% gels, blotted and immunoblotted with a V-type H^+^-ATPase antibody (kindly provided by Margaret Clarke) preferentially reacting with the 37/42 kD subunits of the proton pump.

### Other measurements

cAMP-induced Ca^2+^-influx was measured as described [7]. The centrifugation time of the cells (LvsA^- ^and DH1) during every washing step was shortened without any loss of cells. Protein concentrations were determined with the Coomassie protein assay reagent (Pierce) using bovine serum albumin as standard. Endosomal content was determined by fluid phase uptake of RITC-dextran as described, except that labeling was performed in phosphate buffer pH 6.0 [18].

## Abbreviations

AA: arachidonic acid; BHQ: 2,5-di(tert-butyl)-1,4-hydroquinone; CV: contractile vacuole; CMA: concanamycin A; ER: endoplasmic reticulum; Tg: thapsigargin

## Authors' contributions

DM performed the organelle experiments and in collaboration with CS and DFL designed the experiments and wrote the manuscript. DFL measured receptor-mediated Ca^2+^-influx and responses of living cells to AA; CS cultured the strains and prepared the figures. ADL contributed the strains LvsA^- ^and DH1 and participated in the preparation of the manuscript as did AMT, who also contributed the GFP-strains and Figure 1.
